# Global trade statistics lack granularity to inform traceability and management of diverse and high-value fishes

**DOI:** 10.1038/s41598-017-12301-x

**Published:** 2017-10-09

**Authors:** Donna-Mareè Cawthorn, Stefano Mariani

**Affiliations:** Ecosystems & Environment Research Centre, School of Environment & Life Sciences, Peel Building, The Crescent, University of Salford, Greater Manchester, M5 4WT UK

## Abstract

Illegal, unreported and unregulated (IUU) fishing and seafood supply chain fraud are multifaceted problems that demand multifaceted solutions. Here, we investigate the extent to which global fisheries trade data analyses can support effective seafood traceability and promote sustainable seafood markets using one of the world’s most highly prized, yet misunderstood, groups of fishes as a model: the snappers, family Lutjanidae. By collating and comparing production, import and export data from international and national statistical collections for the period 2006–2013, we show that official trade data severely lack the level of detail required to track snapper trade flows, uncover potential IUU activities and/or inform exploitation management of snappers and related species. Moreover, we contend that the lack of taxonomic granularity and use of vague generic names in trade records represent one of the most insidious impediments to seafood traceability, and suggest that widely used harmonised commodity classification systems should evolve to address these gaps.

## Introduction

Against a backdrop of stagnating marine capture fisheries, global seafood consumption is on the rise and fish has transitioned into one of the world’s most heavily traded food commodities^1^. External market forces dictate the directions of seafood trade flows, and ultimately drive harvest levels, yet the same forces also fuel the illegal behaviour of unscrupulous operators. About one-fifth of world fishery catches are products of illegal, unreported and unregulated (IUU) fishing^2^ – a global scourge that threatens marine ecosystems and the recovery of overexploited fish stocks, fleeces the economy, jeopardises livelihoods, and has frequent links to drug smuggling, human trafficking, and other transnational crimes^3–5^. As seafood trade routes become longer and more complex, there is thus an ever-increasing need to trace seafood from source to consumption, as well as to understand where IUU fishing occurs and how and where illegal products infiltrate the market^3^. The last two decades have consequently seen an overall increase in national ‘responsible fisheries management’ legislation, with a growing number of seafood supply chains adopting advanced technologies (e.g. barcodes, radio frequency identification tags, interoperable software systems, DNA-based tracebacks) and/or opting for voluntary chain-of-custody certification (e.g. component of Marine Stewardship Council [MSC] certification) in pursuit of end-to-end traceability^6,7^. However, developing countries, which contribute over half of global seafood exports^1^, often suffer from weak governance, fragmented and poorly enforced traceability regulations, and limited capacity to ‘police’ their vast swathes of ocean, all of which open doors for unlawful conduct^6^. Moreover, sophisticated technologies and certification may prove prohibitively costly for small-scale fisheries^8^, or be conceivably unattractive to those knowingly operating illegally, implying that independent means are required to investigate illicit harvesting and trade.

Trade data analyses, conducted by comparing existing production, import and export statistics, represent an important ancillary tool for monitoring fisheries-related activities and improving the efficacy of management actions^9^. More specifically, comparisons of trade volumes with the total allowable catch (TAC) and/or reported production volumes of a species can provide insights into the total extraction of that resource and the extent of IUU takings. For instance, Russian crab imports reported by China, Japan, South Korea and the United States (US) between 2000–2012 were found to be two- to four-fold higher than Russia’s officially reported legal crab catch, raising concerns on the levels of illegal harvests and the sustainability of several Russian crab species^10^. Corresponding analyses have suggested substantial IUU catches of Patagonian toothfish (*Dissostichus eleginoides*)^11^ and bluefin tuna (*Thunnus thynnus*)^12^, leading to stricter management measures for these fisheries (e.g. improved surveillance, catch documentation schemes). Furthermore, discrepancies between import and export statistics may indicate circumvention of official trade controls and illuminate ‘hotspots’ for the disposal of IUU product. Such comparisons have uncovered significant incongruities in relation to sea cucumber (*Isostichopus fuscus*) exports from Ecuador^13^, shark exports from South Africa^14^, *T*. *thynnus* traded via Panama^15^, as well as laundering of poached South African abalone (*Haliotis midae*) via neighbouring landlocked countries^13^.

Theoretically, similar trade data analyses should be amendable to many other commercial seafood products, since over 200 countries/territories use the ‘Harmonized System’ (HS) as the basis for national customs tariff codes, which covers *ca*. 98% of global trade. Administered by the World Customs Organization (WCO), the HS comprises over 5,000 commodity codes that are internationally harmonised at the six-digit (HS-6) level. Individual countries are permitted to assign two or more additional digits according to their own tariff and statistical requirements. Although the HS holds potential for monitoring fisheries trade, its nomenclature is rarely standardised to the genus- or species-level^16^. Consequently, numerous commercially important fish may elude monitoring via HS when they are lumped under generic categories that cannot be disaggregated into smaller, distinct taxonomic units^17^. Practical issues may additionally arise in reconciling and comparing country-specific customs data classified at the non-harmonised eight- or 10-digit levels^12^.

The snappers, comprising 112 species and 17 genera in the family Lutjanidae^18^, are one of the world’s most highly prized and important circumtropical marine fisheries resources. Members of the family represent major resources for industrial, artisanal and recreational fisheries, despite several life-history traits that render many vulnerable to overfishing (see Supplementary Fig. S1). With few exceptions, snapper fisheries worldwide are poorly managed and data poor, particularly in the small-scale multi-species fisheries in developing countries (see Supplementary Table S1). Furthermore, although a variety of these species fetch high prices on global markets, there is little methodology in place to trace snappers to their source fisheries, or to monitor the volumes and values entering international trade. Indeed, failings in traceability systems within ‘snapper’ supply chains are evidenced by the frequent reports of substitutions and mislabelling involving these species^19–21^. The US Presidential IUU Task Force recently declared ‘snapper’ as a ‘high risk’ species for IUU fishing and market fraud^22^, and called for enhancements to traceability measures and the specificity of US harmonised tariff codes to better identify such species in trade^23^. This comes as little surprise considering that up to 77% of products sold in the US as ‘red snapper’ have been found to be substituted with other species^24^. Moreover, in 2011, 10–20% of snapper imports into the US from Mexico, as well as 35–50% of imports from Indonesia, were estimated to be from IUU sources^3^. In Indonesia’s Arafura Sea, over 90% of demersal fishes (predominantly snappers) harvested in the longline fishery between 1980 and 2005 were defined as unreported, while 5% were illegally caught^25^.

**Figure 1:**
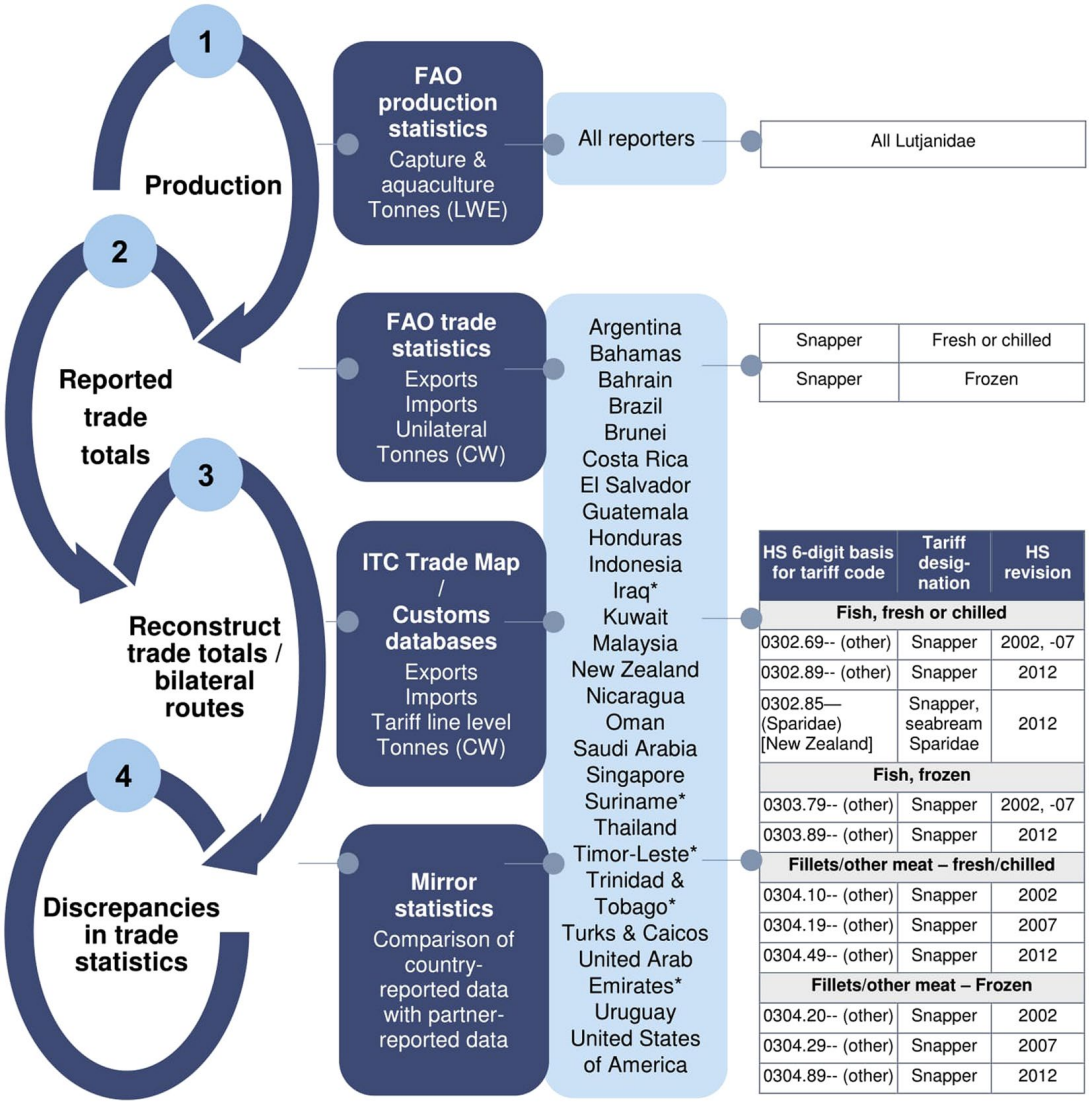
Study design. Overview of the sequential steps taken in collating and comparing global snapper production and trade statistics for the period 2006–2013, including the primary data sources, reporter countries, commodities and HS-6 bases of the trade tariff codes evaluated. A detailed account of individual customs databases and tariff codes consulted for each reporter country is provided in Supplementary Table S2. Reporter countries marked with asterisks are those for which tariff-level trade data could not be sourced and only FAO estimates were available. t = tonnes; LWE = live weight equivalent; CW = commodity weight; ITC = International Trade Centre.

Given the notorious opacity in the worldwide exploitation of snappers, we conducted the first comprehensive global trade data analysis for the family for the period 2006–2013, with the core goals of establishing the primary sources of snapper supply and demand, illuminating existing loopholes that promote trade data discrepancies and facilitate illicit harvesting and trade, and identifying ‘key nodes’ to enhance data reporting in support of more effective traceability. Using official statistics compiled and harmonised by the Food and Agriculture Organization (FAO), we analysed annual and aggregate production volumes for all Lutjanidae spp. for the study period, by country and taxonomic classification^26^. Since trade reported by the FAO^27^ is unidirectional, we reconstructed aggregate snapper trade totals and bilateral trade flows based primarily on national/territorial customs data, with a focus on volumes and commodities recorded under extensions of HS headings 0302 (‘fresh or chilled fish’), 0303 (‘frozen fish’) and 0304 (‘fish fillets and other meat’) (Fig. 1 and Supplementary Table S2). Lastly, to assess the quality of snapper trade statistics and map discrepancies in reported imports and exports, we employed a ‘mirror’ statistics approach^28,29^ to compare country-reported and partner-reported bilateral trade volumes.

## Results and Discussion

### Data caveats

The collation and comparison of snapper production and trade statistics presented several challenges requiring mention, as uncertainties in the analysis almost exclusively stem from data gaps. Firstly, the quality of the data reported by the FAO is largely contingent on the quality of that data provided by its member countries. While it is acknowledged that FAO reported fishery landings often underestimate takings from IUU, subsistence and recreational fishing^30^, one of the objectives of trade data analyses is to assist in approximating the magnitude of this unreported catch^12^. Secondly, the lack of standardised HS-6 commodity codes for snappers precluded the use of the widely employed United Nations Commodity Trade Statistics Database (UN Comtrade, http://comtrade.un.org) for reconstructing snapper trade totals and establishing bilateral trade routes, necessitating a heavy reliance on tariff-line level data available in national/territorial customs databases for this purpose. Such data were often unavailable, poorly accessible, incomplete, non-harmonised, based on different measurement conventions, and/or presented in various dialects. We attempted to account for these gaps by cross-checking data via different sources, and through comparisons with FAO trade totals^27^. Our reconstructed snapper trade totals showed a high degree of congruence with FAO ones, indicating that the method employed for data reconciliation was robust, or at least that this largely accounted for reported trade.

### Main snapper producers

**Figure 2:**
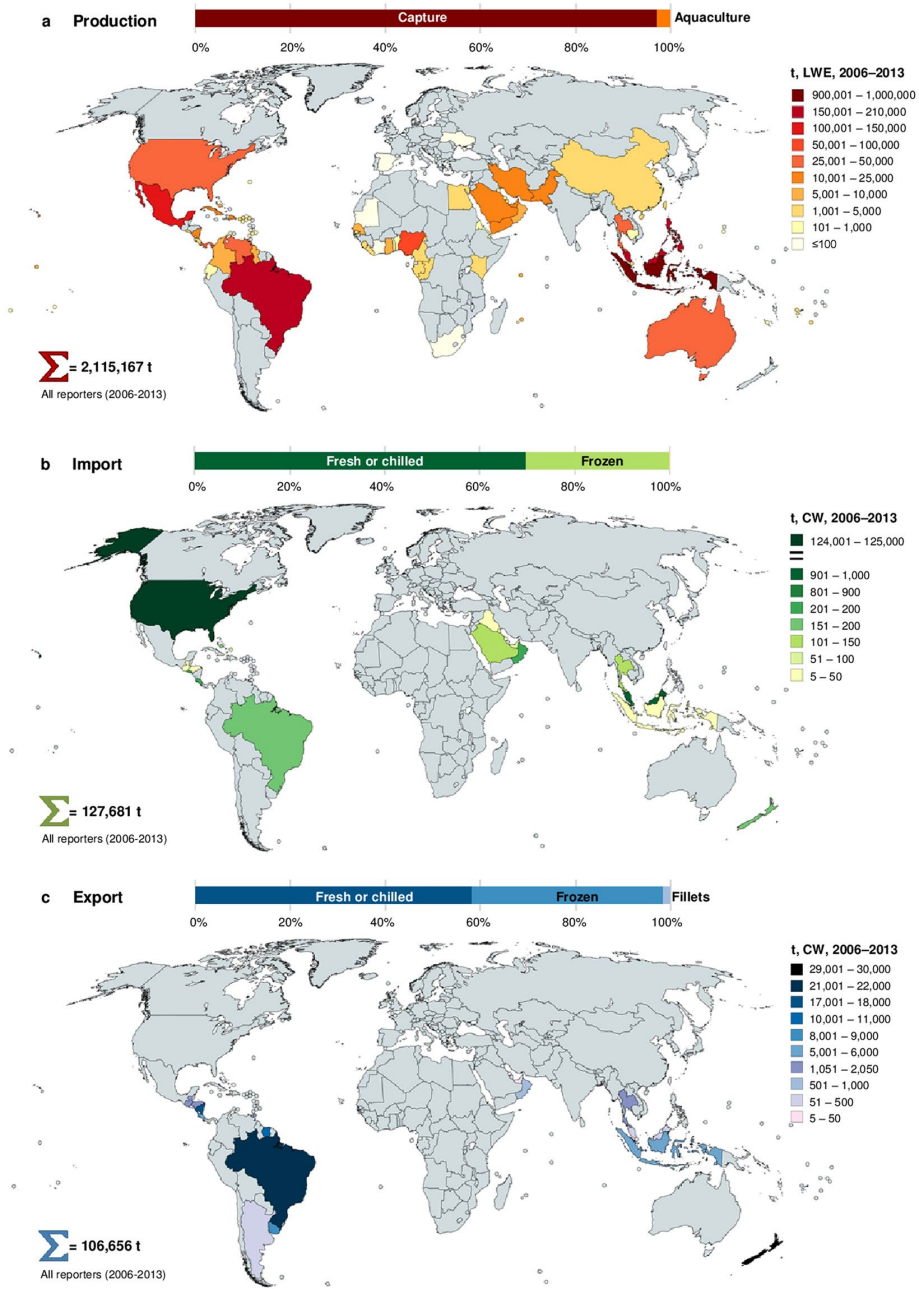
Global reported production and trade totals. (**a** to **c**) Volumes are summed for the period 2006–2013 for (**a**) total reported production of Lutjanidae spp. in t LWE, (**b**) reported snapper imports in t CW, and (**c**) reported export (and re-export) in t CW. Colours transition from dark to light with descending volumes. Production volumes were derived from FAO statistical collections. Snapper trade totals were reconstructed from FAO and national/territorial customs databases and include ‘fresh/chilled fish’ of HS heading 0302, ‘frozen fish’ of HS 0303, and ‘fillets/other meat’ of HS 0304. Countries/territories reporting aggregate production, import or export totals of <5 t over the study period are not indicated. Maps were created using MapChart software, Version 08/2017 (https://mapchart.net).

According to FAO statistics^26^, global aggregate production of Lutjanidae spp. over the study period amounted to ≈2.1 million tonnes (t, live weight equivalent, LWE) (Fig. 2a), with a relatively stable mean annual output of 264,396 t LWE. Approximately 97% of the aggregate supply was derived from capture fisheries, whereas only 3% came from aquaculture. The geographical spread of Lutjanid production (Fig. 2a) generally reflects Lutjanid species-richness patterns (see Supplementary Fig. S1a), with Asia contributing most of the global supply (69%), followed by the Americas (23%), Africa (6%) and Oceania (2%). Indonesia was the single largest Lutjanid-producing country over the study period (45% of global supply), followed by Malaysia (10%), Brazil (7%), the Philippines (7%) and Mexico (5%), while a further 76 countries/territories reported smaller harvests (Fig. 2a).

**Figure 3:**
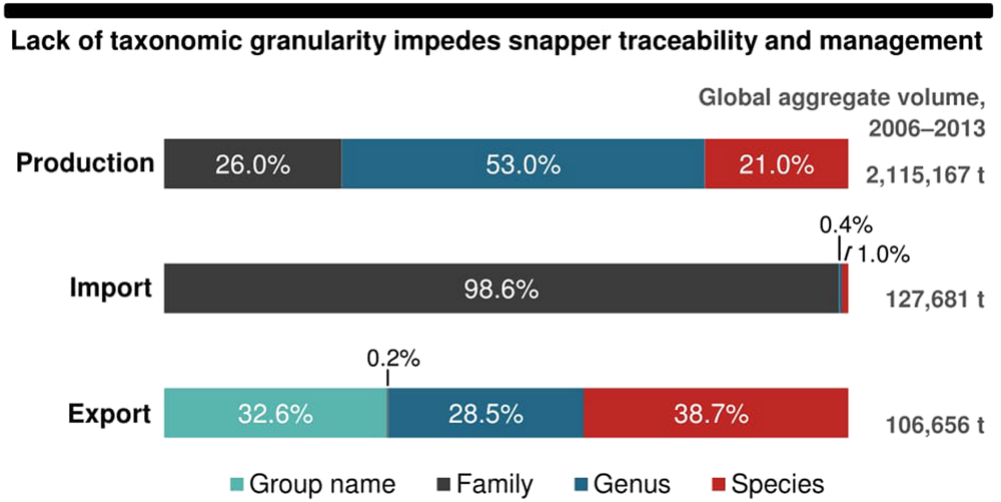
Proportional taxonomic classification levels applied to snappers in global production and trade statistics. ‘Group name’ refers to classifications reported in non-taxonomic terms (e.g. ‘snapper’, ‘pargos’). Data represent aggregated volumes (2006–2013) collated from FAO statistical collections and national/territorial customs databases. Production volumes are in t LWE. Import and export volumes are in t CW, and include ‘fresh/chilled fish’ of HS heading 0302, ‘frozen fish’ of HS 0303, and ‘fillets/other meat’ of HS 0304.

Despite the commercial significance of snappers and their differing vulnerabilities to overfishing (see Supplementary Fig. S1), we observed poor taxonomic resolution in official Lutjanid production records (Fig. 3). A mere 21% of global reported landings were species-specific, with mangrove red snapper (*Lutjanus argentimaculatus*), yellowtail snapper (*Ocyurus chrysurus*), southern red snapper (*L. purpureus*), John’s snapper (*L. johnii*) and northern red snapper (*L. campechanus*) making up the largest part^26^. The overwhelming bulk (79%) of global Lutjanid production was, however, reported only to the genus (snappers nei – *Lutjanus* spp.) or family (snappers, jobfishes nei – Lutjanidae) levels in official records. In part, the reporting of these ‘lumped’ snapper landings may be attributed to the difficulties in identifying individual species in the multi-species fisheries of some countries (e.g. Indonesia), where there is a wide diversity of species with similar morphological features^31,32^. Nonetheless, this paucity of knowledge on species-specific extractions is of concern, given that the structure and status of many snapper stocks in these multi-species fisheries remains unknown, and because harvesting in these fisheries is frequently unsustainable and unregulated (see Supplementary Table S1).

### Main trade reporters

**Figure 4:**
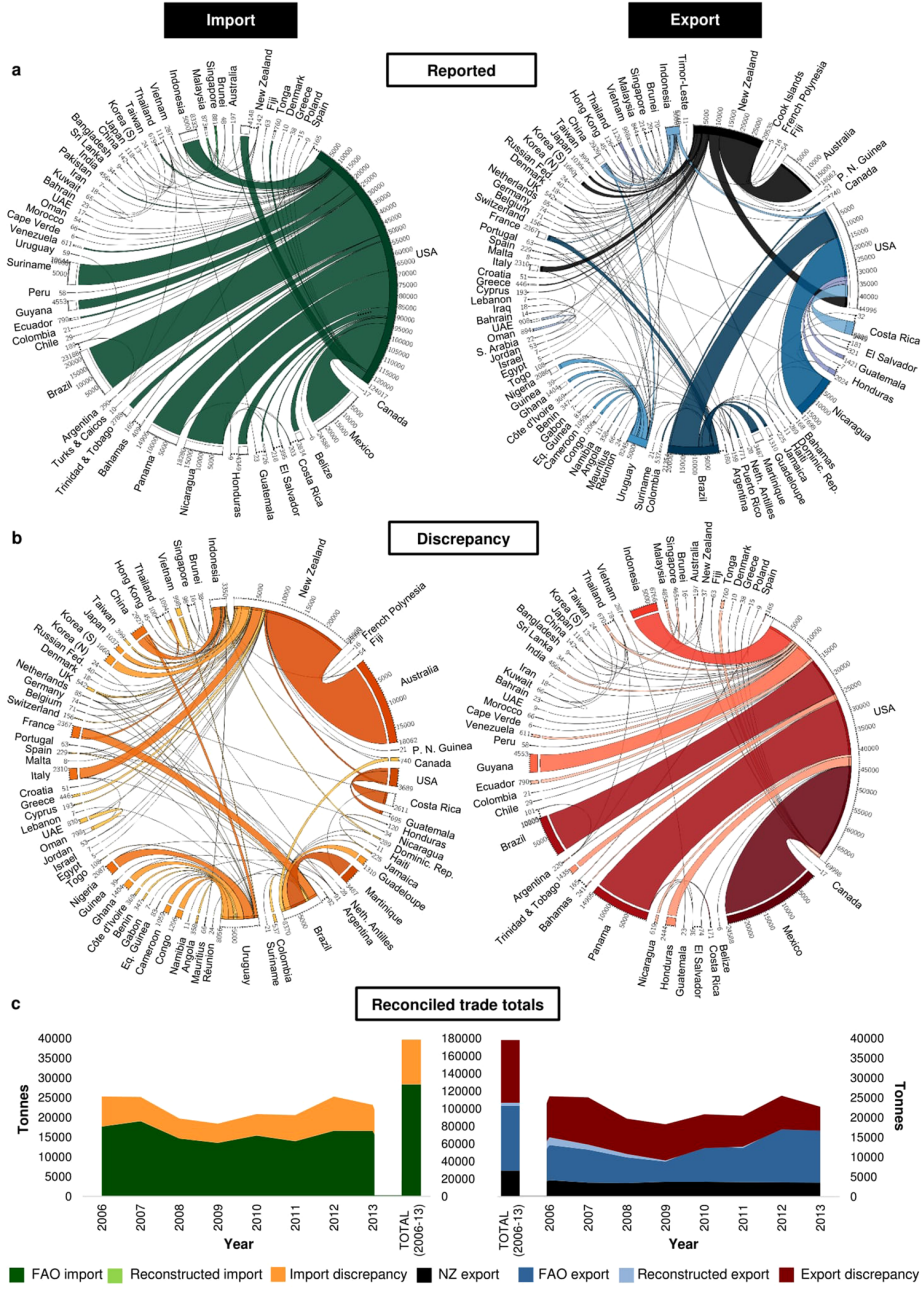
Reported and ‘hidden’ dimensions of the global snapper trade. (**a** to **c**) (**a**) Bilateral trade flows based on reported imports and exports, where the width of bands represents aggregate volumes (t CW) for 2006–2013. Segments of reporter countries touch and have the same colour as bands, while segments of partner countries do not touch bands and are not coloured. Bilateral trade flows summing to <5 t are not indicated. (**b**) Discrepant trade flows revealed through bilateral ‘mirror’ comparisons, where the width of bands represents aggregate volumes (t CW) for 2006–2013. Here, segments of reporter countries are not coloured, while segments of countries that under-reported or did not itemise snappers in trade records touch and have the same colour as bands. Discrepant trade flows summing to <5 t are not indicated. (**c**) Reconciliation of reported and discrepant trade volumes for each year from 2006–2013 and overall. Data are from FAO and customs databases and include ‘fresh/chilled fish’ of HS heading 0302, ‘frozen fish’ of HS 0303, and ‘fillets/other meat’ of HS 0304. NZ = New Zealand; UAE = United Arab Emirates; USA = United States of America.

Comparison of reported import and export totals with production volumes in Fig. 2 indicates that snapper trade is minor relative to production, and this pattern remains even after converting traded commodity weights (CW) to live weights (see Supplementary Fig. S2a). This suggests that the large majority of Lutjanid production is domestically consumed, and/or that trade is underestimated due to non-reporting or aggregation under ‘umbrella’ tariff classifications. Nonetheless, the global trade in snappers appears to be heavily skewed towards the US (Fig. 2b). This country accounted for 97% of reported snapper imports (124,050 t CW) for 2006–2013, and when combined with domestic production, had the world’s largest snapper supply (see Supplementary Fig. S2a). Imports are, however, dominant in the US snapper market, with the majority entering the south-eastern US (i.e. Florida)^33^. Most of these imports comprise ‘fresh or chilled’ fish, whereas the comparatively lower-value ‘frozen’ commodities make up a smaller proportion (Fig. 2b and Supplementary Fig. S3). Mid-range retailers and restaurants in the US are important outlets for imported snapper, whereas higher-end outlets tend to prefer domestically-harvested snapper due to sustainability issues^32^. Although US import records specify at least 60 trading partners for the study period, the bulk of imports were derived from Mexico, Brazil, Nicaragua, Panama and Suriname (10–20%), with Indonesia and Guyana contributing to a lesser extent (5–10%), and all other trade partners providing <5% of the supply (Fig. 4a). The species composition of US snapper imports remains unclear, since the customs records assessed for 2006–2013 were documented only to the family level (i.e. Lutjanidae) (Fig. 3). However, evaluation of imports per US customs area^33^ implies that snappers from Central and South America are favoured in the south-eastern US, likely due to a greater familiarity with the species from these regions (i.e. similar species to those domestically harvested in the south-eastern US)^32^. Imports from elsewhere in the world are more likely to feed into other parts of the US^33^, where there is less familiarity with specific species.

Comparison of reported snapper exports with production volumes (Fig. 2a and c) suggests that the main snapper producers are not necessarily the main exporters, and in some cases no exports are reported for entire continents (i.e. Africa). The world’s largest snapper export reporters for 2006–2013 included New Zealand (29,546 t CW), Brazil (21,304 t CW) and Nicaragua (17,703 t CW) (Fig. 2c). New Zealand’s exports were reportedly distributed to Australia, the US, Europe and Asian countries (Fig. 4a). Central and South American snapper exports followed a consistent trajectory to the US, whereas smaller quantities trickled into the Caribbean and European markets, and Uruguay emerged as the principal supplier of African countries (Fig. 4a). Considering their leading roles as snapper producers, it is worth noting that aggregate exports from Indonesia and Malaysia (Fig. 2c) are likely considerable underestimates, since snapper export records for both countries could only be sourced from customs and/or FAO databases for 2012–2013. Nonetheless, within this short period, Indonesian exports were reportedly split between the US, various Asian countries, and to a lesser extent, European countries (Fig. 4a).

### Discrepancies between production and trade volumes

A given country’s exports of a commodity are not expected to exceed its combined production and imports of that commodity, and where such discrepancies arise, one or more of these records are likely flawed. We discovered inconsistencies with aggregate exports from Guatemala, Honduras, Uruguay and Suriname, which coincide with zero or minimal associated production and imports (Fig. 2a–c and Supplementary Fig. S2a). Exports from Costa Rica and Nicaragua further appear to exceed combined production and import volumes when converted to a live-weight basis (Fig. 2a–c and Supplementary Fig. S2a). Perhaps the most striking anomaly arising in this study relates to the substantial snapper exports reported by New Zealand (29,546 t CW for 2006–2013) (Fig. 2c), given that the island nation reported zero Lutjanid production and minimal snapper imports (151 t CW) for the study period (Fig. 2a and b, Supplementary Fig. S2a). In addition, the native range of Lutjanid species (see Supplementary Fig. S1a) does not appear to extend to the exclusive economic zone (EEZ) of New Zealand proper, although it may well extend to territories in the Realm of New Zealand^34^. It is well known that the silver seabream (*Pagrus auratus*), which is caught in considerable quantities in New Zealand waters (aggregate production, 2006–2013 = 50,514 t LWE)^26^, is often locally referred to as ‘snapper’ despite its placement in the family Sparidae rather than in Lutjanidae^35^. As such, the use of the generic term ‘snapper’ in New Zealand tariff line descriptions could effectively permit the grouping of fish from multiple families in official trade statistics. In 2012, New Zealand amended their trade tariff code descriptions for ‘fresh or chilled’ fish commodities to indicate ‘seabream (Sparidae), snapper’ and ‘seabream (Sparidae), other than snapper’^36^. Yet, exports recorded under the former classification continued to be lumped under ‘snappers’ in the FAO’s trade database^27^. Moreover, exports reported under the amended classifications did not differ substantially in terms of quantity or destination compared with those merely reported as ‘snappers’ in previous years. It is thus likely that New Zealand ‘snapper’ exports comprise partially or exclusively of Sparids, not only skewing domestic trade statistics for the latter family, but also inducing up to a 28% overestimation in true snapper exports reported to the FAO for 2006–2013. Confounding matters further, New Zealand’s ‘snapper’ exports were distributed to at least 46 different destinations over the analysis period (Fig. 4a), potentially distorting the import statistics of partner countries.

### Discrepancies between import and export volumes

In theory, commodity import and export declarations should align closely, as one country’s imports are another country’s exports^12,28^. Comparison of reported snapper import totals (Fig. 2b) and export totals (Fig. 2c), however, suggest that this trade is measured very imperfectly. Aggregate reported imports for 2006–2013 amounted to 127,681 t CW, and averaged 15,960 t CW/annum. Conversely, reported exports for the analysis period summed to 106,656 t CW, averaged 13,332 t CW/annum, and were not significantly correlated with annual reported imports (*R* ^2^ = 0.37, see Supplementary Fig. S4a). Undoubtedly, the lack of harmonised 6-digit codes for Lutjanidae spp. enables such imperfect reporting, since snappers must be classified under generic ‘fish – other’ HS-6 categories. While individual customs offices may add supplementary digits to generic codes to enumerate snappers, others may group snappers with other species or families (see Supplementary Table S2), or not indicate them at all. Nevertheless, import statistics are generally considered to be more reliable than export statistics, since the former must be recorded in sufficient detail to allow customs administrations to apply duties, taxes and additional regulatory controls^28^.

The comparison of country-reported and partner-reported bilateral snapper trade volumes (i.e. ‘mirror’ statistics) forms the basis for the discrepancy charts in Fig. 4b. These charts provide an indication of the ‘hidden’ trade in snappers that is not reflected in official import or export statistics. It is important to highlight that discrepancies in mirror data can arise for various legitimate and illegitimate reasons. Time lags between exports and imports, human errors, and differences in partner country attributions and/or commodity classifications constitute legitimate discrepancies, whereas transhipment, re-export, document falsification, and intentional commodity or direction misclassification are known techniques for disguising trade in illegal goods and/or evading taxes, tariffs, quotas or embargos^10,28,29^. We identified a total of 256 different bilateral snapper trade relationships over the study period, of which only 22 had data reported by both partner countries, while the remainder had data missing on one side of the mirror. Moreover, the analysis revealed >60 countries/territories that did specifically itemise snappers in their customs records. The most obvious reason for the missing data and discrepant volumes (Fig. 4b) lies with the lack of snapper-specific HS-6 codes, and the consequent facilitation of generic trade data reporting. Inevitably, this gap in precision makes it exceedingly difficult to pinpoint and determine the extent to which other factors, including illegal activities, contribute to snapper trade discrepancies.

In terms of inbound trade (Fig. 4b), Australia, as well as African, European and East Asian countries, did not report snapper imports to the FAO for 2006–2013, and no snapper-specific customs records could be sourced reflecting this trade. Several Atlantic/Caribbean islands either did not report snapper imports, or grouped these into non-specific categories alongside other species (see Supplementary Table S2). Additionally, US-reported imports were lower than US-destined exports reported by Costa Rica, Guatemala and New Zealand (Fig. 4b). In terms of outbound trade, major discrepancies emerged due to the absence of reported exports from Mexico (24,588 t CW) and Panama (14,905 t CW), while smaller discrepancies were associated with missing data for Guyana, Ecuador, Venezuela and Tonga (Fig. 4b). US-destined exports from Brazil, Honduras, and Trinidad and Tobago for 2006–2013 were approximately 50% lower than US-reported imports from these countries. Due to the abstruse nature of the snapper supply chain, it was not possible to identify the country(ies) responsible for these discrepancies. The asymmetry in Indonesian exports can likely be attributed to the lack of reported trade data for the country for 2006–2011. It is, however, noteworthy that at least a portion of Indonesia’s and Mexico’s snapper exports to the US during the study period were predicted by Pramod *et al*.^3^ to be from IUU sources.

Global import discrepancies for 2006–2013 summed to 51,037 t CW (mean = 6,342 t CW/annum), whereas global export discrepancies for the study period summed to 71,287 t CW (mean = 8,911 t CW/annum). When adding discrepant values to reported imports and exports, we observed an approximate matching of aggregate trade totals at 178,000 t CW (Fig. 4c) (mean ≈ 22,000 t CW/annum), as well as a highly significant correlation (*R* ^2^ = 0.99) between adjusted annual imports and exports (see Supplementary Fig. S4b). Thus, by reconstructing trade totals and adding discrepant mirror volumes, we show that snapper imports and exports reported to the FAO^27^ for 2006–2013 are underestimated by up to 40% and 73%, respectively. Within the aforementioned ‘matched’ trade total, a volume of *ca*. 29,000 t CW remains linked with New Zealand trade (Fig. 4c) and associated taxonomic uncertainty, excluding which, trade totals likely level at *ca*. 149,000 t CW (mean ≈ 18,600 t CW/annum) for the study period. Nevertheless, achieving consistency in trade totals does not necessarily assure credible trade flows. Mirror statistics, although better than no data at all, fail to account for trade between two non-reporting countries, which may prove especially problematic for developing countries that lack the expertise and technologies for handling trade data^29^.

## Conclusions and Policy Recommendations

In many regions of the world, snappers are being exploited without much systematic rigour and transparency. Yet, it is impossible to develop effective management and conservation plans without knowing the magnitude of exploitation and demand, as well as understanding which species are involved. Informative trade-based estimations of bluefin tuna (*T*. *thynnus*) catches and levels of IUU harvests^12^ have previously been possible because unique HS-6 codes exist for this species, permitting uniform reconciliation of reported import and export data and comparison to *T*. *thynnus* quotas and reported landings. Other trade data analyses that have suggested considerable IUU takings of toothfish^11^ and abalone^13^ have shortly been followed by the designation of genus-level HS-6 codes for these taxa. Here, we have demonstrated that the lack of snapper-specific HS-6 codes allows for a considerable portion of trade to go undocumented, while the generic terms used in tariff line descriptions enables the inclusion of non-Lutjanids and the distortion of global snapper trade statistics. We therefore conclude that, based on existing commodity classification systems, it is not possible to accurately track the volumes of true snapper in trade – not to the family level, and certainly not to the species level. Moreover, given the ambiguities associated with this poor HS-code specificity, no concrete inferences can be drawn on the extent to which dubious activities contribute to snapper trade data discrepancies, nor on the potential magnitude of illegal snapper harvests (or unreported harvests in quota-absent fisheries). While our study focused on snappers, a similar scenario is likely to arise with other valuable and highly-exploited fish for which no HS-6 codes are currently reserved (e.g. groupers, orange roughy – *H. atlanticus*).

We acknowledge that the HS was originally developed for tariff purposes, but believe that the system represents an underutilised and useful tool for monitoring and controlling various aspects of fishery (and other wildlife) trade. We therefore support the call by Chan *et al*.^17^ to standardise HS-codes to the 10-digit level, allowing for the listing of thousands of commodities per sub-heading. Standardisation of the codes to this extent could provide for direct cross-verification of regional trade data, improved detection of illegal trade, and a more robust basis for enforcement actions. If such an approach is not adopted, we suggest that the WCO consider assigning unique six-digit HS codes to the family Lutjanidae. To justify the creation of such sub-headings, the annual global trade of a commodity should exceed US$50 million^17^. The annual reported export value of ‘fresh or chilled’ snapper alone exceeded this threshold in both 2012 and 2013^27^, even when excluding the value of discrepant trade uncovered in this study. Designation of family-specific HS-6 codes would potentially allow customs offices to use the subsequent two digits to enumerate the main Lutjanid species in trade, while reserving further digits for individual customs or statistical requirements. This improvement in taxonomic granularity should aid both traceability and fisheries management, since it is ‘species’ that are targets of fishing and ‘species’ that must be protected from overexploitation. As the precarious state of global fisheries resources brings an ever-increasing need for information, we contend that strengthening the HS would be an important step forward in meeting broader fisheries traceability and sustainability targets.

## Methods

### Study Design

Snapper production and trade statistics were collated and compared for an eight-year period (2006–2013), using various data sources detailed below and in Supplementary Table S2. This analysis period was selected because, at the time of analysis (July–December 2016), the FAO Fishery Commodities and Trade statistical collection^27^ included snapper import and export records only up to and including 2013, and several national/territorial customs databases only began reporting snapper trade data after 2006. The results are aggregated for the study period and for examined commodities, unless otherwise specified.

### Production statistics

As the only repository for global fisheries data, the FAO’s publicly-available fisheries production statistics (see Supplementary Table S2) served as the main point of comparison with available trade data. Annual and aggregate snapper production volumes (t LWE) were derived from the online ‘Global Production’ database^26^, with the relative contributions of capture fisheries and aquaculture being evaluated in the individually-collated FAO databases (see Supplementary Table S2). Data were filtered by selecting i) all reporters under the ‘country’ search tab, ii) all species listed within the family Lutjanidae under the ‘taxonomic classification’ search tab, and iii) the years 2006 to 2013 under the ‘time’ search tab. Aggregate production volumes for the British Virgin Islands, Cambodia, Cameroon, the Cook Islands, Fiji, Gabon, Hong Kong SAR, Kiribati, Liberia and Yemen included FAO estimates for some or all of the years^26^. Countries were ranked based on Lutjanid production volumes for the eight-year period, with the results being visualised using MapChart software (https://mapchart.net).

### Trade statistics

Snapper trade data were derived from various sources for different purposes (see Supplementary Table S2), with a focus on the traded volumes (t) of three main commodity groups (‘fresh or chilled fish’, ‘frozen fish’ and fresh or frozen ‘fish fillets and other meat’). Although trade values (US$) were additionally recorded, the different valuations applied to imports (Cost, Insurance, Freight – CIF) and exports (Free on Board – FOB) introduce variability in price data and potentially bias direct comparisons of bilateral trade flows.

#### FAO baseline trade data

The FAO’s Fishery Commodities and Trade database^27^ served as a starting point to assess global snapper supply and demand dynamics, as well as the main actors engaged in snapper trade. This database contains annual import and export (and re-export) records by volume (t CW) and value (thousand US$) for ‘snappers – fresh or chilled’ and ‘snappers – frozen’, corresponding to HS headings 0302 and 0303, respectively. Nonetheless, the FAO database does not contain disaggregated data for snapper ‘fillets and other meat’, corresponding to HS heading 0304, which likely leads to underestimations in the FAO’s snapper trade totals. Moreover, since trade reported by the FAO is unidirectional (i.e. no information is provided on the origin of reported imports or the destinations of reported exports), more detailed investigations were required to elucidate the bilateral exchanges underpinning the global snapper trade network.

#### Reconstructed trade totals and bilateral flows

Preliminary explorations of snapper trade flows, key trading partners and sources of national/territorial customs data were conducted in Trade Map (www.trademap.org), an online market analysis tool maintained by the International Trade Centre (ITC, http://www.intracen.org). Data were accessed at the tariff-line level for 2006–2013 using the ‘advanced search’ option within Trade Map, selecting ‘all countries’ and inputting a variety of keywords to capture all relevant records (i.e. ‘snapper’, ‘pargos’, ‘vivaneau’, ‘huachinango’, ‘Lutjanidae, ‘Apsilinae’, ‘Etelinae’, ‘Paradicichthyinae’, ‘*Lutjanus*’, ‘*Etelis*’, ‘*Aphareus*’, ‘*Aprion*’, ‘*Apsilus*’, ‘*Hoplopagrus*’, ‘*Lipocheilus*’, ‘*Macolor*’, ‘*Ocyurus*’, ‘*Pinjalo*’, ‘*Paracaesio*’, ‘*Parapristipomoides*’, ‘*Pristipomoides*’, ‘*Randallichthys*’, ‘*Rhomboplites*’, ‘*Symphorichthys*’, ‘*Symphorus*’). Retrieved records were carefully filtered to remove duplicates arising from the keyword search and to exclude tariff-line listings that grouped ‘snappers’ with other fish types (see Supplementary Table S2). Whenever possible, relevant national/territorial customs databases indicated as data sources in Trade Map were accessed directly, recording import, export and re-exports at the tariff-line level (extensions of HS 0302, 0303 and 0304), as indicated in Supplementary Table S2 Import and export (and re-export) records were subsequently synthesised into separate datasets, including the year, reporter country, associated country of destination (exports) or origin (imports), trade volume (t CW) and trade value (thousand US$). Using this approach, bilateral trade routes were tracked for 99% of aggregate ‘fresh or chilled’ and ‘frozen’ snapper import volumes reported by the FAO for 2006–2013, and when adding sourced customs data for snapper ‘fillets and other meat’, our reconstructed aggregate snapper import totals exceeded the FAO’s aggregate import volumes by 0.2%. In terms of exports and re-exports, the reconstructed aggregate totals (2006–2013) for ‘fresh or chilled’ and ‘frozen’ snapper were 1% higher than FAO aggregate volumes (excluding FAO estimates), and when adding sourced export data for snapper ‘fillets and other meat’, this discrepancy increased to 3% (see Fig. 4c). Country-specific FAO estimates, for which bilateral trade routes could not be established, were added back to reconstructed imports or exports (as appropriate) to estimate the trade totals. Global aggregate trade volume maps were constructed using MapChart software (https://mapchart.net), while the circular data figures showing bilateral trade flows were generated with Circos software^37^. These bilateral trade depictions do not attempt to match snapper commodities that were imported only to be subsequently exported (re-exports), although FAO data suggest that such re-exports comprised a very small proportion of total snapper exports (487 t CW for 2006–2013, equivalent to <0.5% of all exports documented for this period)^27^.

#### Estimating trade data discrepancies

The aforementioned bilateral trade flows were based on reported imports and exports, but closer examination of these datasets revealed incongruities between some country-reported and partner-reported trade totals, as well as between the absolute number of reporter countries and specified trading partners. A reciprocal or ‘mirror’ statistics approach was therefore employed to estimate discrepancies arising from under- or non-reported snapper trade, comparing a given importer’s imports with its trading partner’s exports, and vice versa. The following two equations were utilised to measure such discrepancies (t CW), in which (i) the reporting countries are importers, and (ii) the reporting countries are exporters:

i $$Di{f}_{ct}^{ij}=E{x}_{ct}^{ij}-I{m}_{ct}^{ij}$$

where *Ex* indicates partner *j* reported exports of commodity *c* from country *i* at year *t*, whereas *Im* indicates reporting country *i* reported imports of commodity *c* at the same time period *t*.

ii $$Di{f}_{ct}^{ij}=I{m}_{ct}^{ij}-E{x}_{ct}^{ij}$$

where *Im* indicates partner *j* reported imports of commodity *c* from country *i* at year *t*, whereas *Ex* indicates reporting country *i* reported exports of commodity *c* at the same time period *t*.

Mirror discrepancies were summed across all examined commodities for bilateral trade partners, and were also summed to provide net global import and export discrepancies on both an annual and aggregate basis. It is important to note that these discrepancies do not necessarily signify illegal trade, since reporter countries may well have reported snappers at higher levels of aggregation or under non-specific commodity codes (e.g. ‘fish – other’). Nonetheless, they provide an indication of the volume of traded fish that is not itemised as snapper and a more accurate depiction of the true magnitude of the global snapper trade.

### Reconciling production and trade volumes

To better compare snapper production and trade volumes, and estimate country-specific supplies, we identified the top snapper producers, importers and exporters for 2006–2013, and applied a suite of conversion factors to harmonise traded commodity weights with live weights. Between capture and trade, snappers may be gutted, gilled, dressed, filleted or otherwise processed. Suitable conversion factors must be applied in accordance with the level of processing when approximating live weight. For ‘fresh or chilled’ and ‘frozen’ snapper commodities, we used snapper-specific conversion factors of 1.14 for ‘gutted, head on’ commodities^38^ and 1.65 for ‘gutted, head off’ commodities^39^. When tariff line descriptions of ‘fresh or chilled’ and ‘frozen’ commodities did not expressly indicate the processing state, we applied an average of the latter two factors, i.e. 1.40. A snapper-specific conversion factor of 3.12 was used for ‘fillets’^38^. Converted product weights were summed across all commodities for 2006–2013 to estimate total traded volumes (in LWE) for each country. We acknowledge that, given the estimations and assumptions applied in conversions, these ‘live weight’ calculations may not be perfect, but are likely to offer a better comparison with production volumes than commodity weights.

### Data availability

Additional materials, data and protocols not included in the manuscript or supporting information will be made available to requesting readers by the corresponding author in material transfer agreements.

## Electronic supplementary material

Supplementary information
