# Publisher Correction: Global trade statistics lack granularity to inform traceability and management of diverse and high-value fishes

**DOI:** 10.1038/s41598-017-16205-8

**Published:** 2017-11-17

**Authors:** Donna-Mareè Cawthorn, Stefano Mariani

**Affiliations:** Ecosystems & Environment Research Centre, School of Environment & Life Sciences, Peel Building, The Crescent, University of Salford, Greater Manchester, M5 4WT UK

*Scientific Reports* **7**:12852; doi:10.1038/s41598-017-12301-x; Article published online 09 October 2017

The original HTML version of this Article contained an error in the Results and Discussion under subheading ‘Main trade reporters’.

“Comparison of reported snapper exports with production volumes (Fig. 2a and c) suggests that the main snapper producers are not comment = “Please change to ‘necessarily’ “necessarily the main exporters, and in some cases no exports are reported for entire continents (i.e. Africa)”.

now reads:

“Comparison of reported snapper exports with production volumes (Fig. 2a and c) suggests that the main snapper producers are not necessarily the main exporters, and in some cases no exports are reported for entire continents (i.e. Africa)”.

A correction to this article has been published and is linked from the HTML version of this paper. The error has been fixed in the paper.

