# Supplementary material for: Global trade statistics lack granularity to inform traceability and management of diverse and high-value fishes
Source: Sci Rep. 2017 Oct 9;7:12852. doi: 10.1038/s41598-017-12301-x (PMC5634443; doi:10.1038/s41598-017-12301-x)
Supplement: Supplementary file 1 — Supplementary information [file 41598_2017_12301_MOESM1_ESM.pdf]

SUPPLEMENTARY INFORMATION

TITLE: Global trade statistics lack granularity to inform traceability and management of diverse and high-value fishes

AUTHORS:

Donna-Mareè Cawthorn[1]*, Stefano Mariani[1]

[1]Ecosystems & Environment Research Centre, School of Environment & Life Sciences, Peel Building, The Crescent, University of Salford, Greater Manchester, M5 4WT, UK.

***Correspondence to:**

Donna-Mareè Cawthorn

Ecosystems & Environment Research Centre, School of Environment & Life Sciences, Peel Building, The Crescent, University of Salford, Greater Manchester, M5 4WT, UK.

Tel: +44 74 2486 5614

E-mail: d.m.cawthorn@salford.ac.uk, cawthorndonna@gmail.com

Content:

Table S1-S2

Figures S1-S4

Supplementary Table S1. Quality of management and stock status of global snapper fisheries. Data are derived from FishSource (www.fishsource.org), a program of Sustainable Fisheries Partnership (SFP).

Supplementary Table S2. Snapper production and trade data used in this study. Trade data include customs tariff codes and descriptions of snapper commodities used in tariff line headings, where applicable. Tariff codes that were excluded from the analysis are also shown.

FishSource scores are calculated on a scale from zero to 10, with 10 reflecting the best-case scenario. Fisheries are further ranked into three sustainability categories (A, B, C) based on FishSource management scores (1-3) and stock status scores (4-5), where:
Category A – Very well managed fisheries scoring ≥8 across all five FishSource scores;
Category B – Reasonably managed fisheries ≥6 across all five FishSource scores;
Category C – Poorly managed fisheries with at least one FishSource score <6.

Name	Scientific name	Management unit	Flag country	Fishing gear	Category	Management quality (1-3)			Stock health (4-5)		Fishery Improvement Programs (FIPS)	Certification (Marine Stewardship Council)	Notes
						1. Management strategy	2. Managers compliance	3. Fishers compliance	4. Current status	5. Future status			
Snappers nei	*Lutjanus* spp.	Southern Java to Western of Timor Sea (WPP-573)	Indonesia	Bottom-set longlines Handlines hand operated	No score	No score	No score	No score	No score	No score	Aru, Arafura and Timor Seas snapper and grouper - handlines, bottom set longline: Stage 4, Progress Rating B	None	Multi-species fishery. Main snappers caught are *Lutjanus malabaricus* and *L. erythropterus*. No consensus on stock structure of these species. Snapper fisheries within the region are fished by thousands of fishers with multiple gears, including large industrial-scale multi species vessels and many subsistence fishers. Collection of data and information is challenging. Stock assessment for *L. malabaricus* and *L. erythropterus* conducted between 1999-2003. Current fishing levels on snapper stocks in the regions shown to be unsustainable. IUU fishing remains rampant in Aru, Arafura and Timor Seas.
				Handlines mechanized	No score	No score	No score	No score	No score	No score	None	None	
Snappers nei	*Lutjanus* spp.	Aru Bay, Arafuru Sea and Eastern of Timor Sea (WPP-718)	Indonesia	Bottom-set longlines Handlines hand operated	C	<6	No score	<6	No score	<6	Aru, Arafura and Timor Seas snapper and grouper - handlines, bottom set longline: Stage 4, Progress Rating B	None	
				Handlines mechanized	C	<6	No score	<6	No score	<6	None	None	
Snappers nei	*Lutjanus* spp.	Java Sea (WPP-712)	Indonesia	Bottom-set longlines Handlines hand operated Hooks & lines	C	<6	No score	No score	<6	No score	None	None	Snappers in Java Sea considered overexploited. Catch reporting system is deficient. Stock and exploitation status of specific species unknown. Information on stock structure lacking. No management strategy known to be in place for this fishery. Small-scale fisheries in Indonesia are practically unregulated, without control over fishing capacity.
Snappers nei	*Lutjanus* spp.	Tomini Bay - Seram Seas (WPP-715)	Indonesia	Bottom-set longlines Handlines hand operated	No score	No score	No score	No score	No score	No score	Indonesia Snapper and Grouper: Stage 4, Progress Rating C	None	Multi-species fishery. No consensus on stock structure. No species-specific stock assessments.
Snappers nei	*Lutjanus* spp.	Tolo Bay and Banda Sea (WPP-714)	Indonesia	Bottom-set longlines Handlines hand operated	No score	No score	No score	No score	No score	No score	Indonesia Snapper and Grouper: Stage 4, Progress Rating C	None	Multi-species fishery. No consensus on stock structure. No species-specific stock assessments.
Snappers nei	*Lutjanus* spp.	Makassar Strait - Flores Sea (WPP-713)	Indonesia	Bottom-set longlines Handlines hand operated	C	<6	No score	No score	≥6	No score	Indonesia Snapper and Grouper: Stage 4, Progress Rating C Snapper-Grouper Makassar Strait: Stage 4, Progress Rating B	None	Snappers in the region are mainly fished by small-scale fishery, which is an unregulated and unreported fishery. A comprehensive nation-wide biological stock assessment for snappers (*Lutjanus* spp.) is lacking.
Snappers nei	*Lutjanus* spp.	Makassar Strait - Flores Sea (WPP-713)	Indonesia	Gillnets and entangling nets Hooks & lines	C	<6	No score	No score	≥6	No score	Snapper-Grouper Makassar Strait: Stage 4, Progress Rating B	None	

Jobfishes nei	*Pristipomoides* spp.	Southern Java to Western of Timor Sea (WPP-573)	Indonesia	Bottom-set longlines Handlines hand operated	No score	No score	No score	No score	No score	No score	Aru, Arafura and Timor Seas snapper and grouper - handlines, bottom set longline: Stage 4, Progress Rating B	None	
				Handlines mechanized	No score	No score	No score	No score	No score	No score	None	None	
Jobfishes nei	*Pristipomoides* spp.	Aru Bay, Arafuru Sea and Eastern of Timor Sea (WPP-718)	Indonesia	Bottom-set longlines Handlines hand operated	No score	No score	No score	No score	No score	No score	Aru, Arafura and Timor Seas snapper and grouper - handlines, bottom set longline: Stage 4, Progress Rating B	None	
				Handlines mechanized	No score	No score	No score	No score	No score	No score	None	None	
Jobfishes nei	*Pristipomoides* spp.	Java Sea (WPP-712)	Indonesia	Bottom-set longlines Handlines hand operated Hooks & lines	No score	No score	No score	No score	No score	No score	None	None	Multi-species fishery. Not clear if individual jobfish species are assessed by Indonesia's Commission for Stock Assessments
Jobfishes nei	*Pristipomoides* spp.	Tolo Bay and Banda Sea (WPP-714)	Indonesia	Bottom-set longlines Handlines hand operated	No score	No score	No score	No score	No score	No score	Indonesia Snapper and Grouper: Stage 4, Progress Rating C	None	
Jobfishes nei	*Pristipomoides* spp.	Tomini Bay - Seram Seas (WPP-715)	Indonesia	Bottom-set longlines Handlines hand operated	No score	No score	No score	No score	No score	No score	Indonesia Snapper and Grouper: Stage 4, Progress Rating C	None	
Jobfishes nei	*Pristipomoides* spp.	Makassar Strait - Flores Sea (WPP-713)	Indonesia	Bottom-set longlines Handlines hand operated	No score	No score	No score	No score	No score	No score	Indonesia Snapper and Grouper: Stage 4, Progress Rating C	None	
			Indonesia	Gillnets and entangling nets Hooks & lines	No score	No score	No score	No score	No score	No score	None	None	
Crimson jobfish	*Pristipomoides filamentosus*	Indonesia	Indonesia	Hooks and lines	No score	No score	No score	No score	No score	No score	None	None	
Emperor red snapper	*Lutjanus sebae*	Makassar Strait - Flores Sea (WPP-713)	Indonesia	Bottom-set longlines Handlines mechanized	No score	No score	No score	No score	No score	No score	None	None	
Emperor red snapper	*Lutjanus sebae*	Andaman Sea	Thailand	Bottom trawls Handlines mechanized	No score	No score	No score	No score	No score	No score	None	None	
Emperor red snapper	*Lutjanus sebae*	Gulf of Thailand	Thailand	Bottom trawls Handlines mechanized	No score	No score	No score	No score	No score	No score	None	None	
Snappers, jobfishes nei	Lutjanidae	Vietnam	Vietnam	Handlines hand operated	No score	No score	No score	No score	No score	No score	None	None	
Grey snapper	*Lutjanus griseus*	US Gulf of Mexico	United States	Handlines hand operated Hooks & lines	No score	No score	No score	No score	No score	No score	None	None	Stock structure not completely understood.
Northern red snapper	*Lutjanus campechanus*	US Gulf of Mexico	United States	Handlines hand operated Longlines	C	7.4	10	10	3.6	9.4	None	None	Assessment completed in 2013 indicated that stock remains overfished, but is not experiencing overfishing and is rebuilding.

Vermilion snapper	*Rhomboplites aurorubens*	US Gulf of Mexico	United States	Vertical Lines	A	≥8	10	10	8.6	10	None	None	Stock assessment in 2011 indicated that stock is not overfished or undergoing overfishing.
Mutton snapper	*Lutjanus analis*	US Gulf of Mexico<br>US Southern Atlantic	United States	Hooks & lines<br>Longlines	No score	No score	No score	No score	No score	No score	None	None	
Yellowtail snapper	*Ocyurus chrysurus*	US Gulf of Mexico<br>US NW Atlantic	United States	Hooks & lines	A	≥8	10	≥8-10	10	10	None	None	Stock structure not clearly understood. Most recent stock assessment (2012) indicated that population is not overfished or experiencing overfishing.
Northern red snapper	*Lutjanus campechanus*	Mexico	Mexico	Hooks & lines	No score	No score	No score	No score	No score	No score	None	None	
Silk snapper	*Lutjanus vivanus*	Gulf of Mexico	Mexico	Hooks & lines<br>Longlines	No score	No score	No score	No score	No score	No score	None	None	No available information on stock structure
Yellowtail snapper	*Ocyurus chrysurus*	Southern Gulf of Mexico	Mexico	Hooks & lines	C	<6	<6	<6	No score	No score	None	None	Stock status of yellowtail snapper in Mexican waters unknown. Snappers considered to be "in deterioration" in Yucatán, Campeche and Veracruz and "at the sustainable maximum" in Tamaulipas, Quintana Roo and Tabasco. Illegal fishing detected in Natural Park Arrecife Alacranes, as well as in National Park Arrecifes de Cozumel.
Spotted rose snapper	*Lutjanus guttatus*	Costa Rica<br>Eastern Pacific	Costa Rica	Bottom-set longlines	No score	No score	No score	No score	No score	No score	None	Nicoya Peninsula artisanal snapper: Withdrawn July 2016	
Lane snapper	*Lutjanus synagris*	Costa Rica	Costa Rica	Hooks and lines	No score	No score	No score	No score	No score	No score	None	None	Stock structure of lane snapper in Costa Rica waters unknown.
Yellowtail snapper	*Ocyurus chrysurus*	Honduras<br>Western Central Atlantic	Honduras	Hooks and lines	No score	No score	No score	No score	No score	No score	None	None	Stock structure not clearly understood.
Silk snapper	*Lutjanus vivanus*	Nicaragua<br>Western Central Atlantic	Nicaragua	Hooks & lines<br>Longlines	C	<6	<6	<6	<6	<6	None	None	No available information on structure or status of stock in Nicaraguan waters. Nicaraguan fisheries troubled with legislation violations, undersized catches, fishing during closed seasons, poor catch data reporting. Available information suggests moderate to high by-catch levels in snapper fisheries.
Lane snapper	*Lutjanus synagris*	Nicaragua<br>Western Central Atlantic	Nicaragua	Longlines	No score	No score	No score	No score	No score	No score	None	None	Stock structure of Lane snapper in Nicaragua waters unknown.
Lane snapper	*Lutjanus synagris*	Panama	Panama	Longlines	No score	No score	No score	No score	No score	No score	None	None	Stock structure of lane snapper in Panama waters unknown.

Southern red snapper	*Lutjanus purpureus*	Brazil	Brazil	Hooks & lines Traps	C	≥6	<6	<6	<6	<6	North Brazilian Caribbean red snapper - hook/line and pot/trap: Stage 3, Progress Rating E	None	A single population appears to exist in Brazilian waters. Stock in a poor condition and overexploited for years.
Yellowtail snapper	*Ocyurus chrysurus*	Brazil	Brazil	Handlines hand operated Pole-lines hand operated	No score	No score	No score	No score	No score	No score	None	None	
Southern red snapper	*Lutjanus purpureus*	Guyana	Guyana	Traps	No score	No score	No score	No score	No score	No score	None	None	Stock structure of southern red snapper in Caribbean region poorly understood.
Southern red snapper	*Lutjanus purpureus*	Suriname	Suriname	Hooks and lines	No score	No score	No score	No score	No score	No score	None	None	

COUNTRY	DATA SOURCE	TARIFF CODES	DESIGNATION
**Production statistics**			
**All countries**	FAO total global production statistics.<br>FAO global capture production statistics.<br>FAO global aquaculture production statistics.<br>[Accessed via online query panels, www.fao.org/fishery/topic/16140/en].	NA	Lutjanidae
**Trade statistics (import, export, re-export)**			
**Argentina**	2006-2013: National Institute of Statistics and Censuses (INDEC, www.indec.gov.ar). [Accessed via the INDEC foreign trade query system (https://comex.indec.gov.ar/search)].	0302.69.23	Snappers
		0303.79.33	Snappers
		0304.20.20	Snappers (fillets, frozen)
		0304.29.20	Red snappers (fillets, frozen)
**Bahamas**	2007-2013: Bahamas Department of Statistics (www.bahamas.gov.bs/statistics).<br>2006: International Trade Centre's (ITC's) Trade Map (www.trademap.org) statistics.	0302.69.40	Snapper (Lutjanidae)
		0302.89.40	Snapper (Lutjanidae)
		0303.79.40	Snapper (Lutjanidae)
		0303.89.40	Snapper (Lutjanidae)
		0304.10.30	Snapper (fillets, fresh/chilled)
		0304.19.30	Snapper (fillet/other meat, fresh/chilled)
		0304.49.30	Snapper (fillet, fresh/chilled)
		0304.20.30	Snapper (fillets, frozen)
		0304.29.30	Snapper (fillets, frozen)
		0304.89.30	Snapper (fillets, frozen)
**Bahrain**	2006-2013: International Trade Centre's (ITC's) Trade Map (www.trademap.org) statistics.	0303.79.30	Snappers, jobfishes (Lutjanidae)
		0303.89.30	Red snappers
**Brazil**	2007-2013: Brazil Foreign Trade Secretariat. [Accessed via System of Foreign Trade Information Analysis - ALICEWEB MERCOSUL (www.alicewebmercosul.mdic.gov.br)].<br>2006: Data from Scavage Foreign Trade Statistics (www.scavage.com).	0302.69.23	Southern red snappers
		0302.89.12	Snapper (*Lutjanus purpureus*)
		0303.79.33	Southern red snappers
		0303.89.32	Snapper (*Lutjanus purpureus*)
		0304.20.20	Southern red snappers (fillets, frozen)
		0304.29.20	Southern red snapper (*Lutjanus purpureus*) (fillets, frozen)
**Brunei Darussalam**	Department of Economic Planning and Development (JPKE, www.depd.gov.bn) statistics. [Accessed via the International Trade Centre's (ITC's) Trade Map (www.trademap.org)].	0302.89.18	Mangrove red snappers
		0303.89.18	Mangrove red snappers
**Costa Rica**	2006-2013: Central Bank of Costa Rica and PROCOMER (www.procomer.com) statistics. [Accessed via PROCOMER statistics portal, http://servicios.procomer.go.cr/estadisticas/inicio.aspx].	0302.69.20.00	Snappers (*Lutjanus* spp.)
		0302.89.10.00	Snappers (*Lutjanus* spp.)
		0304.20.20.00	Snapper (fillets, frozen)
		0304.29.10.00	Snappers (*Lutjanus* spp.) (fillets, frozen)
		0304.89.10.00	Snappers (*Lutjanus* spp.) (fillets, frozen)
**El Salvador**	2006-2013: Central Reserve Bank of El Salvador foreign trade database (www.bcr.gob.sv).	0302.89.10	Snapper (*Lutjanus* spp.)
		0304.89.10	Snappers (*Lutjanus* spp.) (fillets, frozen)
**Guatemala**	2006-2013: Central Bank of Guatemala statistics (www.banguat.gob.gt). [Accessed via the International Trade Centre's (ITC's) Trade Map (www.trademap.org)].	0302.69.20	Snapper (*Lutjanus* spp.)
		0302.89.10	Snapper (*Lutjanus* spp.)
		0304.29.10	Snapper (*Lutjanus* spp.) (fillets, frozen)
**Honduras**	2006-2013: National Institute of Statistics (INE) of Honduras (www.ine.gob.hn).<br>[Accessed via the International Trade Centre's (ITC's) Trade Map (www.trademap.org)].	0302.69.20.00	Snapper (*Lutjanus* spp.)
		0302.89.10.00	Snapper (*Lutjanus* spp.)
		0304.20.20.00	Snapper (frozen fillets)
		0304.89.10.00	Snapper (*Lutjanus* spp.) (fillets, frozen)
**Indonesia**	2006-2013: BPS-Statistics Indonesia (www.bps.go.id) statistics. [Accessed via the International Trade Centre's (ITC's) Trade Map (www.trademap.org)].	0302.89.18.00	Mangrove red snappers
		0303.89.18.00	Mangrove red snappers
**Iraq**	2006-2013: FAO trade statistics (**Estimates**) [Accessed via FAO Fishery Commodities and Trade Statistics (www.fao.org/fishery/statistics/global-commodities-production/query/en)].	NA	Snapper (fresh/chilled)
**Kuwait**	2006-2013: State of Kuwait Central Statistical Bureau (www.csb.gov.kw) statistics. [Accessed via the International Trade Centre's (ITC's) Trade Map (www.trademap.org)].	0302.69.30	Snappers, Jobfish (Lutjanidae)
		0302.89.30	Red snappers (Jobfishes)
		0303.79.30	Snappers, Jobfish (Lutjanidae)
**Malaysia**	2006-2013: FAO trade statistics [Accessed via FAO Fishery Commodities and Trade Statistics (www.fao.org/fishery/statistics/global-commodities-production/query/en)].	NA	Snapper (fresh/chilled)
		NA	Snapper (frozen)

**New Zealand**	2006-2013: Statistics New Zealand [Accessed via Infoshare, www.stats.govt.nz/infoshare].	0302.69.01.35	Snapper
		0302.69.11.35	Snapper
		0302.69.19.35	Snapper
		0302.85.00.10	Snapper, seabream (Sparidae)
		0302.85.00.21	Snapper, seabream (Sparidae)
		0302.85.00.31	Snapper, seabream (Sparidae)
		0303.79.01.55	Snapper
		0303.79.11.55	Snapper
		0303.79.19.65	Snapper
		0303.89.10.53	Snapper
		0303.89.20.53	Snapper
		0303.89.90.55	Snapper
		0304.10.00.61	Snapper (fillets/other meat, fresh/chilled)
		0304.19.00.56	Snapper (fillets/other meat, fresh/chilled)
		0304.20.00.67	Snapper (fillets, frozen)
		0304.29.00.57	Snapper (fillets, frozen)
		0304.49.00.57	Snapper (fillets, fresh/chilled)
		0304.89.00.65	Snapper (fillets, frozen)
**Nicaragua**	2006-2013: Directorate General of Customs Services of Nicaragua (www.dga.gob.ni).<br>[Accessed via the International Trade Centre's (ITC's) Trade Map (www.trademap.org)].	0302.69.20.000	Snapper (*Lutjanus* spp.)
		0302.89.10.000	Snapper (*Lutjanus* spp.)
		0304.29.10.000	Snapper (*Lutjanus* spp.) (fillets, frozen)
		0304.89.10.000	Snapper (*Lutjanus* spp.) (fillets, frozen)
**Oman**	2006-2011: FAO trade statistics [Accessed via FAO Fishery Commodities and Trade Statistics (www.fao.org/fishery/statistics/global-commodities-production/query/en)].<br>2012-2013: International Trade Centre's (ITC's) Trade Map (www.trademap.org) statistics.	0302.89.30	Red snappers
		0303.89.30	Red snappers
**Saudi Arabia**	2006-2013: FAO trade statistics [Accessed via FAO Fishery Commodities and Trade Statistics (www.fao.org/fishery/statistics/global-commodities-production/query/en)].	NA	Snapper (frozen)
**Singapore**	2006-2013: International Enterprise Singapore, Ministry of Trade and Industry statistics. [Accessed via the International Trade Centre's (ITC's) Trade Map (www.trademap.org)].	0302.89.18	Mangrove red snappers
		0303.89.18	Mangrove red snappers
**Suriname**	2006-2013: FAO trade statistics (**Estimates**) [Accessed via FAO Fishery Commodities and Trade Statistics (www.fao.org/fishery/statistics/global-commodities-production/query/en)].	NA	Snapper (fresh/chilled)
		NA	Snapper (frozen)
**Thailand**	2007-2013: Customs department of the kingdom of Thailand statistics (http://en.customs.go.th).<br>2006: UN Comtrade Statistics (http://comtrade.un.org).	0302.89.18.000	Mangrove red snappers (*Lutjanus argentimaculatus*)
		0303.89.18.000	Mangrove red snappers (*Lutjanus argentimaculatus*)
**Timor-Leste**	2006-2013: FAO trade statistics (**Estimates**) [Accessed via FAO Fishery Commodities and Trade Statistics (www.fao.org/fishery/statistics/global-commodities-production/query/en)].	NA	Snapper (frozen)
**Trinidad & Tobago**	2006-2013: FAO trade statistics (**Estimates**) [Accessed via FAO Fishery Commodities and Trade Statistics (www.fao.org/fishery/statistics/global-commodities-production/query/en)].	NA	Snapper (fresh/chilled)
		NA	Snapper (frozen)
**Turks & Caicos**	2006-2013: FAO trade statistics [Accessed via FAO Fishery Commodities and Trade Statistics (www.fao.org/fishery/statistics/global-commodities-production/query/en)].	NA	Snapper (fresh/chilled)
		NA	Snapper (frozen)
**United Arab Emirates**	2006-2013: FAO trade statistics (**Estimates**) [Accessed via FAO Fishery Commodities and Trade Statistics (www.fao.org/fishery/statistics/global-commodities-production/query/en)].	NA	Snapper (fresh/chilled)
		NA	Snapper (frozen)
**Uruguay**	2007-2013: Data from UTECEM (Technical Unit of Statistics of Foreign Trade of Mercosur). [Accessed via System of Foreign Trade Information Analysis - ALICEWEB MERCOSUL (www.alicewebmercosul.mdic.gov.br)].<br>2006: Data from Scavage Foreign Trade Statistics (www.scavage.com).	0302.69.23	Snappers (*Lutjanus purpureus*)
		0303.79.33	Southern red snapper
		0303.89.32	Snapper (*Lutjanus purpureus*)
**United States of America**	2006-2013: US Census Bureau statistics (https://usatrade.census.gov). Cross-checked in the International Trade Centre's (ITC's) Trade Map (www.trademap.org) and the National Oceanic and Atmospheric Administration (NOAA) National Marine Fisheries Service (NMFS) foreign trade database (www.st.nmfs.noaa.gov/commercial-fisheries/foreign-trade).	0302.69.40.40	Snapper (Lutjanidae spp.)
		0302.69.50.58	Snapper (Lutjanidae spp.)
		0302.89.50.58	Snapper (Lutjanidae spp.)
		0303.79.00.67	Snapper (Lutjanidae spp.)
		0303.79.40.75	Snapper (Lutjanidae spp.)
		0303.89.00.67	Snapper (Lutjanidae spp.)

Excluded from analysis (import, export, re-export)			
**Antigua & Barbuda**	International Trade Centre's (ITC's) Trade Map (www.trademap.org).	0302.69.20	Snapper, croaker, grouper, dolphin, bangamary & sea trout
		0302.69.20.00	
		0302.69.20.10	
		0302.69.20.90	
		0303.79.20	
		0303.79.20.00	
**Barbados**	International Trade Centre's (ITC's) Trade Map (www.trademap.org).	0302.69.20.90	Snapper, croaker, grouper, dolphin, bangamary & sea trout
		0303.79.20.90	
**Belize**	International Trade Centre's (ITC's) Trade Map (www.trademap.org).	0302.69.20	Snapper, croaker, grouper, dolphin, bangamary & sea trout
		0303.79.20	
		0303.89.20	Snapper, croaker, grouper, dolphinfish, bangamary
**Dominica**	International Trade Centre's (ITC's) Trade Map (www.trademap.org).	0303.79.20	Snapper, croaker, grouper, dolphin, bangamary & sea trout
**Grenada**	International Trade Centre's (ITC's) Trade Map (www.trademap.org).	0302.69.20	Snapper, croaker, grouper, dolphin, bangamary & sea trout
		0303.79.20	
**Guyana**	International Trade Centre's (ITC's) Trade Map (www.trademap.org).	0302.69.20.00	Snapper, croaker, grouper, dolphin, bangamary & sea trout
		0303.79.20.00	
**Jamaica**	International Trade Centre's (ITC's) Trade Map (www.trademap.org).	0302.69.20.00	Snapper, croaker, grouper, dolphin, bangamary & sea trout
		0302.89.20.00	Other: Snapper, croaker, grouper, dolphinfish (mahi-mahi) & bangamary
		0303.79.20.00	Snapper, croaker, grouper, dolphin, bangamary & sea trout
		0303.89.20.00	Other: Snapper, croaker, grouper, dolphinfish (mahi-mahi) & bangamary
**Montserrat**	International Trade Centre's (ITC's) Trade Map (www.trademap.org).	0303.79.20	Snapper, croaker, grouper, dolphin, bangamary & sea trout
**Saint Kitts & Nevis**	International Trade Centre's (ITC's) Trade Map (www.trademap.org).	0302.69.20	Other: Snapper, croaker, grouper, dolphin, bangamary & sea trout
		0303.79.20	
**Saint Lucia**	International Trade Centre's (ITC's) Trade Map (www.trademap.org).	0302.69.20	Snapper, croaker, grouper, dolphin, bangamary & sea trout
		0303.79.20	
		0303.89.20	Other: Snapper, croaker, grouper, dolphinfish (mahi-mahi) & bangamary
**St. Vincent & Grenadines**	International Trade Centre's (ITC's) Trade Map (www.trademap.org).	0302.69.20	Snapper, croaker, grouper, dolphin, bangamary & sea trout
		0303.79.20	
**Trinidad & Tobago**	International Trade Centre's (ITC's) Trade Map (www.trademap.org).	0302.69.20	Snapper, grouper sea trout, dolphin etc.
		0303.79.20	

Supplementary Figure S1. Characteristics of the family Lutjanidae. (**a** to **e**). The number of assessed Lutjanid species according to (a) global species-richness patterns, (b) taxonomic classification, (c) human uses, (d) intrinsic vulnerability to fishing based on scores out of 100, and (e) commercial value. The map in (a) was created in AquaMaps[1]. Taxonomic data in (b) were derived from the Catalog of Fishes[2]. Human use data in (c) were from FishBase[3]. Vulnerability scores in (d) are based on fuzzy logic expert models and were accessed via FishBase[3]. Commercial value data in (e) are based on global ex-vessel fish prices and were accessed via FishBase[3].

Supplementary Figure S2. Snapper supply-and-demand by primary producers and/or traders. (**a** to **b**). Comparisons of country-specific aggregate production, import and export volumes (t LWE) for 2006–2013, as well as estimated overall supply, where (a) is based on reported volumes, and (b) includes both reported volumes and discrepant trade volumes estimated by bilateral 'mirror' data comparisons. Estimated supply in t LWE was calculated as: (production + import) – export. Production volumes were derived from FAO statistical collections. Snapper trade totals were reconstructed from FAO and national / territorial customs databases and include 'fresh / chilled fish' of HS heading 0302, 'frozen fish' of HS 0303, and 'fillets / other meat' of HS 0304. Export volumes for Suriname are based on FAO estimates[4]. Trade commodity weights were expressed as LWE using commodity-specific conversion factors.

Supplementary Figure S3. Snapper imports and exports by commodity. (**a** to **c**). (a) Annual reported trade volumes (t CW) by individual commodity and overall, (b) annual mean price per unit weight (US$ / kg) per commodity, (c) relative contribution of each commodity to total aggregate trade volumes (t CW) for 2006–2013.

Supplementary text: Differences in mean commodity prices (US$ / kg) for imports and exports may be reflective of the different valuations applied to imports (CIF) and exports (FOB).

Supplementary Figure S4. Correlation plots between reported and adjusted import and export volumes (t CW). (**a** to **b**). (a) Lack of association between annual reported snapper import and export volumes (Pearson's $R = 0.61$, $p = 0.11$, and only *ca*. 37% variance explained) across the study period. (b) After adjusting snapper trade totals by adding discrepant mirror volumes to reported trade volumes, the correlation between annual imports and exports becomes near perfect (Pearson's $R = 0.99$, $p << 0.001$).

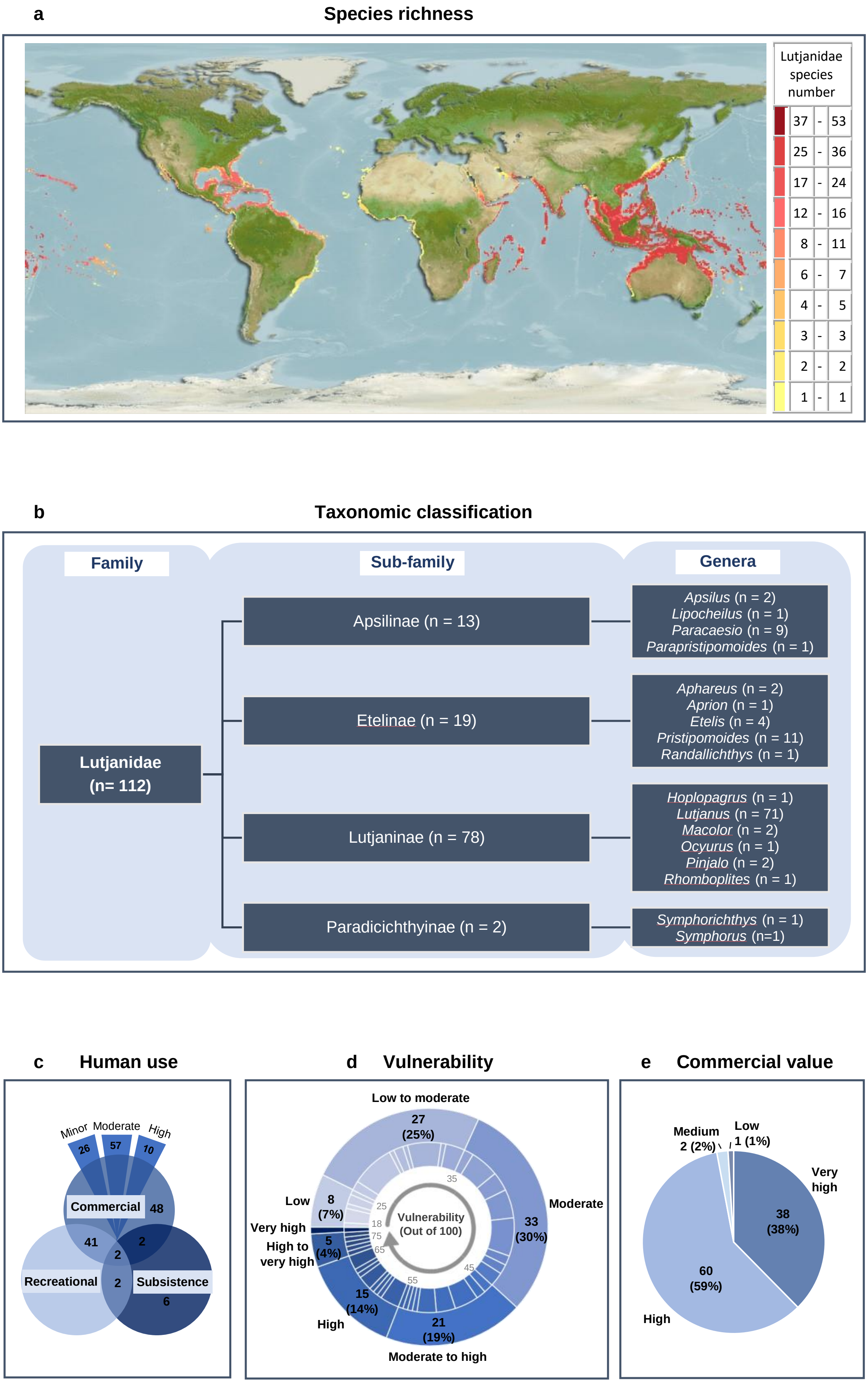
a
Species richness
Lutjanidae species number
37 - 53
25 - 36
17 - 24
12 - 16
8 - 11
6 - 7
4 - 5
3 - 3
2 - 2
1 - 1
b
Taxonomic classification
Family
Sub-family
Genera
Lutjanidae (n= 112)
Apsilinae (n = 13)
Etelinae (n = 19)
Lutjaninae (n = 78)
Paradicichthyinae (n = 2)
Apsilus (n = 2)
Lipocheilus (n = 1)
Paracaesio (n = 9)
Parapristipomoides (n = 1)
Aphareus (n = 2)
Aprion (n = 1)
Etelis (n = 4)
Pristipomoides (n = 11)
Randallichthys (n = 1)
Hoplopagrus (n = 1)
Lutjanus (n = 71)
Macolor (n = 2)
Ocyurus (n = 1)
Pinjalo (n = 2)
Rhomboplites (n = 1)
Symphorichthys (n = 1)
Symphorus (n=1)
c
Human use
Minor
Moderate
High
26
57
10
Commercial
48
41
2
2
Recreational
2
Subsistence
6
d
Vulnerability
Low to moderate
27 (25%)
Moderate
33 (30%)
Moderate to high
21 (19%)
High
15 (14%)
Very high
High to very high
5 (4%)
Low
8 (7%)
Vulnerability (Out of 100)
35
25
18
75
65
55
45
e
Commercial value
Medium 2 (2%)
Low 1 (1%)
Very high
38 (38%)
60 (59%)
High

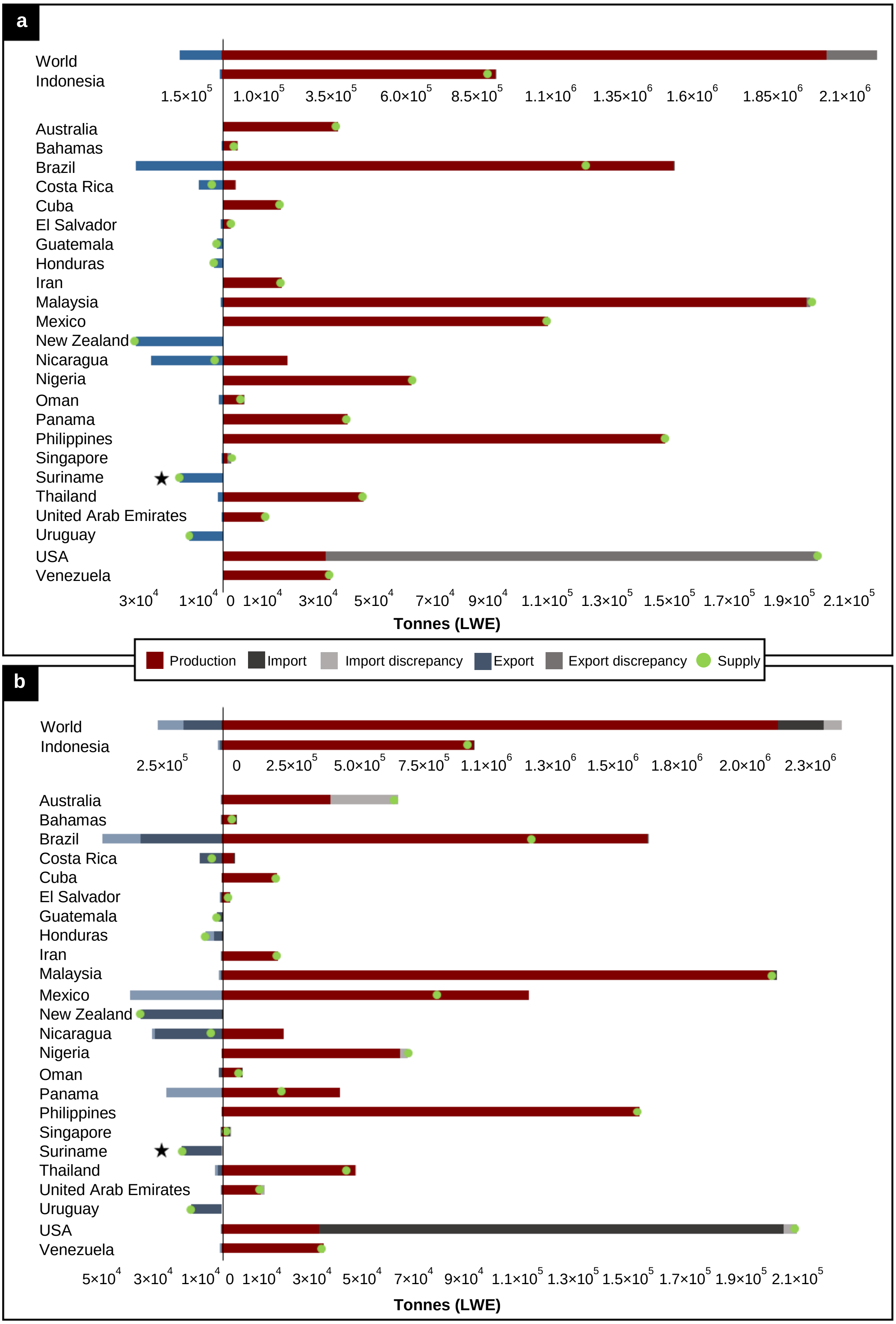
a
World
Indonesia
1.5×10^5 1.0×10^5 3.5×10^5 6.0×10^5 8.5×10^5 1.1×10^6 1.35×10^6 1.6×10^6 1.85×10^6 2.1×10^6
Australia
Bahamas
Brazil
Costa Rica
Cuba
El Salvador
Guatemala
Honduras
Iran
Malaysia
Mexico
New Zealand
Nicaragua
Nigeria
Oman
Panama
Philippines
Singapore
Suriname
Thailand
United Arab Emirates
Uruguay
USA
Venezuela
3×10^4 1×10^4 0 1×10^4 3×10^4 5×10^4 7×10^4 9×10^4 1.1×10^5 1.3×10^5 1.5×10^5 1.7×10^5 1.9×10^5 2.1×10^5
Tonnes (LWE)
Production
Import
Import discrepancy
Export
Export discrepancy
Supply
b
World
Indonesia
2.5×10^5 0 2.5×10^5 5.0×10^5 7.5×10^5 1.1×10^6 1.3×10^6 1.5×10^6 1.8×10^6 2.0×10^6 2.3×10^6
Australia
Bahamas
Brazil
Costa Rica
Cuba
El Salvador
Guatemala
Honduras
Iran
Malaysia
Mexico
New Zealand
Nicaragua
Nigeria
Oman
Panama
Philippines
Singapore
Suriname
Thailand
United Arab Emirates
Uruguay
USA
Venezuela
5×10^4 3×10^4 1×10^4 0 1×10^4 3×10^4 5×10^4 7×10^4 9×10^4 1.1×10^5 1.3×10^5 1.5×10^5 1.7×10^5 1.9×10^5 2.1×10^5
Tonnes (LWE)

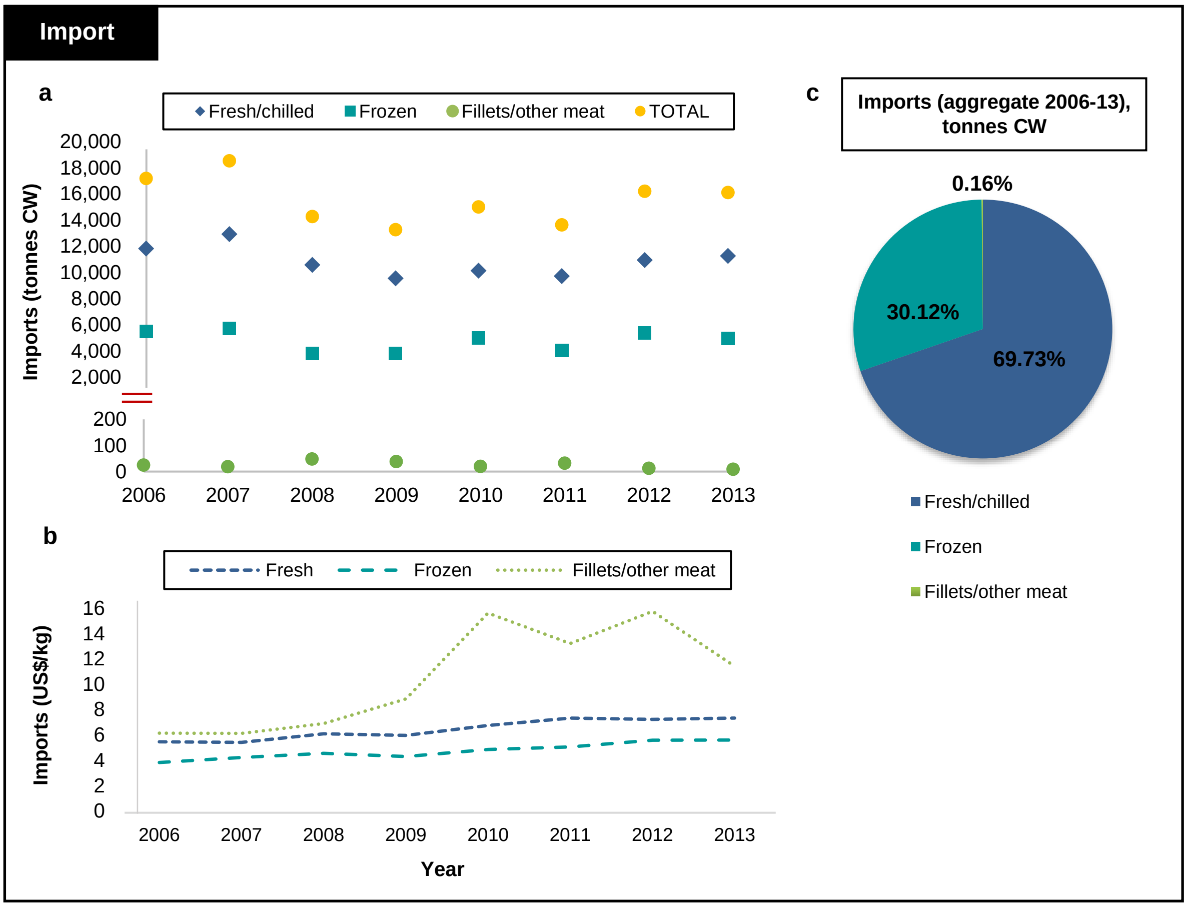
Import
a
Fresh/chilled
Frozen
Fillets/other meat
TOTAL
Imports (tonnes CW)
20,000
18,000
16,000
14,000
12,000
10,000
8,000
6,000
4,000
2,000
200
100
0
2006
2007
2008
2009
2010
2011
2012
2013
b
Fresh
Frozen
Fillets/other meat
Imports (US$/kg)
16
14
12
10
8
6
4
2
0
2006
2007
2008
2009
2010
2011
2012
2013
Year
c
Imports (aggregate 2006-13), tonnes CW
0.16%
30.12%
69.73%
Fresh/chilled
Frozen
Fillets/other meat

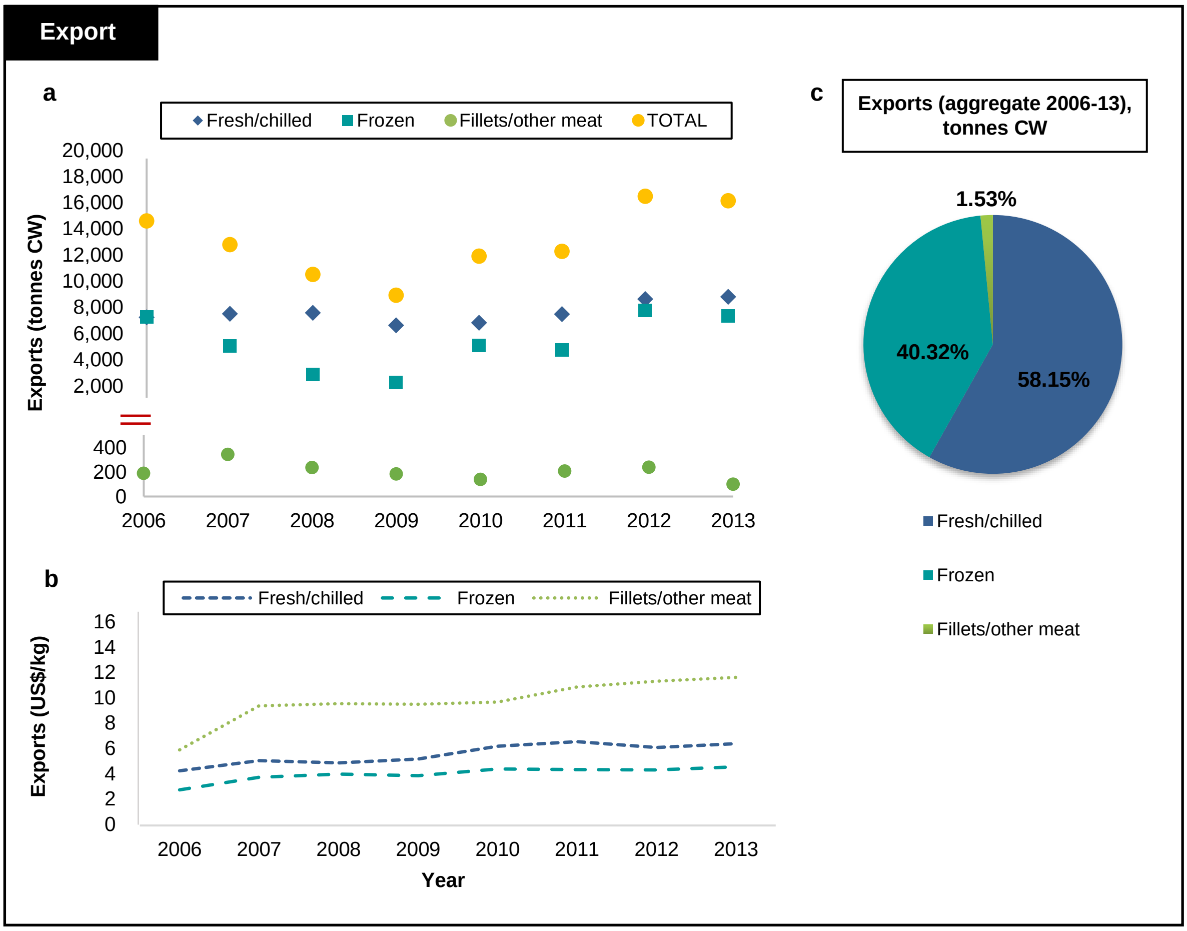
Export
a
Fresh/chilled
Frozen
Fillets/other meat
TOTAL
Exports (tonnes CW)
20,000
18,000
16,000
14,000
12,000
10,000
8,000
6,000
4,000
2,000
400
200
0
2006
2007
2008
2009
2010
2011
2012
2013
b
Fresh/chilled
Frozen
Fillets/other meat
Exports (US$/kg)
16
14
12
10
8
6
4
2
0
2006
2007
2008
2009
2010
2011
2012
2013
Year
c
Exports (aggregate 2006-13), tonnes CW
1.53%
40.32%
58.15%
Fresh/chilled
Frozen
Fillets/other meat

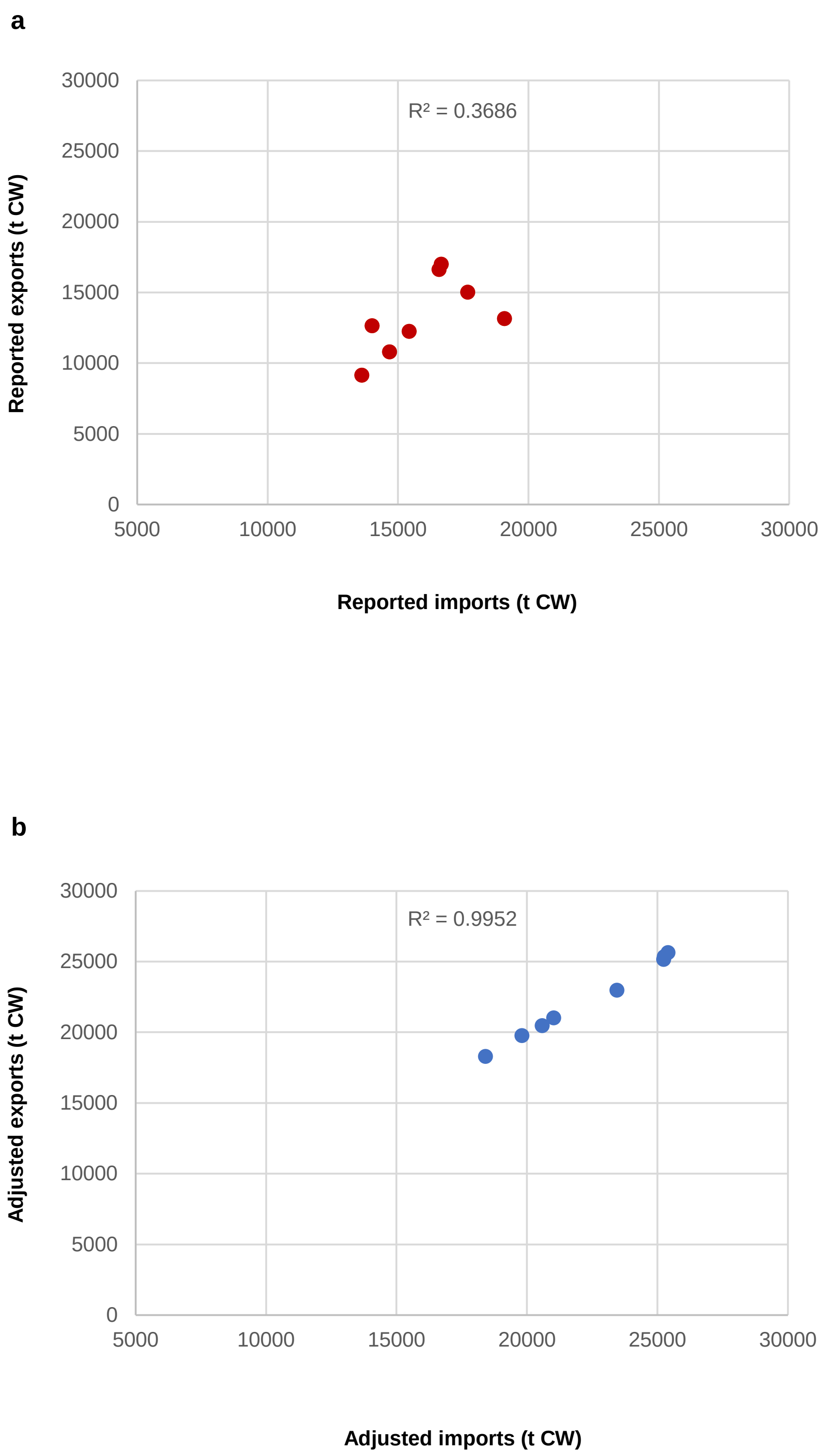
a
R² = 0.3686
Reported exports (t CW)
Reported imports (t CW)
30000
25000
20000
15000
10000
5000
0
5000
10000
15000
20000
25000
30000
b
R² = 0.9952
Adjusted exports (t CW)
Adjusted imports (t CW)
30000
25000
20000
15000
10000
5000
0
5000
10000
15000
20000
25000
30000

REFERENCES

1. Kaschner, K. *et al*. *AquaMaps: Predicted Range Maps for Aquatic species. World Wide Web Electronic Publication, Version 08/2016* www.aquamaps.org (2016). Accessed on 08 August 2017.
2. Eschmeyer, W. N. & Fong, J. D. *Catalog of Fishes: Species by Family/Subfamily* http://researcharchive.calacademy.org/research/ichthyology/catalog/fishcatmain.asp (2017). Accessed on 03 March 2017.
3. Froese, R. & Pauly, D. *FishBase* www.fishbase.org (2017). Accessed on 03 August 2017.
4. Food and Agriculture Organization. *Fishery Commodities and Trade 1976–2013* http://www.fao.org/fishery/topic/16140/en (2017). Accessed on 27 July 2017.
